# Using a Factorial Survey to Estimate the Relative Importance of Well-Being Dimensions According to Older People: Insights From a Repeated Survey Experiment in Flanders

**DOI:** 10.1093/geroni/igac034

**Published:** 2022-05-19

**Authors:** Veerle Van Loon, Koen Decancq

**Affiliations:** Centre for Social Policy Herman Deleeck, University of Antwerp, Antwerp, Belgium; Centre for Social Policy Herman Deleeck, University of Antwerp, Antwerp, Belgium

**Keywords:** Preferences, Response consistency, Temporal reliability, Test–retest analysis

## Abstract

**Background and Objectives:**

Although it has become standard to include the views of older people when assessing their well-being, most existing methods are ill-suited to estimate the relative importance of well-being dimensions. This article investigates the potential of the factorial survey method to estimate the relative importance of six well-being dimensions (health, income, social relations, leisure, engaging activities, and religion) based on the views of older people themselves.

**Research Design and Methods:**

We implemented a factorial survey in a repeated survey experiment among 800 older adults in Flanders (the Dutch-speaking northern part of Belgium). We performed several within-sample test–retests to investigate the consistency of the estimated coefficients over time (i.e., temporal reliability). In addition, we tested the feasibility of the factorial survey by studying two indicators of cognitive load: response time and response consistency.

**Results:**

We estimated the relative importance of increases in six well-being dimensions. Increases from the lowest level to the highest level in the dimensions of health, income, and social relations had the highest effect on well-being—followed by leisure, engaging activities, and religion. The results proved to be consistent in most of the test–retest analyses. Furthermore, we found that respondents produced a high level of response consistency within an acceptable amount of response time.

**Discussion and Implications:**

The findings suggest that the factorial survey method offers a promising way forward to elicit older people’s views on well-being and, hence, in developing tailored policies that matter to them.

**Translational significance**: We investigated the potential of a factorial survey to estimate the relative importance of well-being dimensions among older people. Overall, the estimated importance of the dimensions proved to be plausible and reliable. In addition, we observed a high level of response consistency within an acceptable amount of response time. Consequently, the factorial survey can be considered a valuable survey strategy for policymakers and practitioners interested in evaluating and enhancing the well-being of older people according to their own views.

## Background and Objectives

Over the next three decades, the number of older people is expected to double worldwide, reaching over 1.5 billion in 2050 (United Nations, 2020). This rapid aging and the associated pressure on public health care systems and funding has intensified international interest in the promotion of well-being at an older age (Pruchno, 2015). In this vein, the World Health Organization has recently declared a Decade of Healthy Aging (2020–2030), with its action plan aiming to improve the well-being of older people by putting their experiences and expertise at the center.

In the academic literature, “well-being” is often used as an umbrella term, serving different purposes, and capturing different underlying concepts (Ryff et al., 2021). A large body of work exists on the measurement of the psychological and dynamic notion of well-being, its effect on other social phenomena and the factors that influence it (see, e.g., Lee et al., 2021). In this article, however, we follow the philosophical and public policy-oriented literature to define a well-being measure as an instrument that is constructed to make interpersonal well-being comparisons (Adler & Fleurbaey, 2016; Adler & Norheim, 2022). Such an instrument is indispensable to any theory of justice or public policy with a special concern for the distribution of well-being, for those who are the worst-off or for the most vulnerable in society (Rawls, 1982) and, as such, is essential to evaluate the design of aging policies aimed at enhancing the well-being of older people (Pruchno, 2015).

Inherently, any well-being construct designed to make interpersonal comparisons is value-laden (Robbins, 1935). Not surprisingly, there are different approaches to making interpersonal well-being comparisons, among which the objective, subjective, and preference-based approaches are the most popular (Decancq et al., 2015a). The former approach builds on an “objective list” conception of well-being to construct a composite well-being index. This approach has been criticized for being overly paternalistic (Fleurbaey & Blanchet, 2013) and for providing a conception of well-being that is potentially alienated from the individual’s own perception of well-being (Fletcher, 2015). The subjective approach, on the other hand, relies on measures of subjective well-being such as happiness and life satisfaction to make well-being comparisons (see, e.g., Dolan & Fujiwara, 2016; Layard, 2005). Although this approach has recently gained interest, subjective measures have been criticized for being sensitive to individual idiosyncrasies such as expensive tastes (Arrow, 1973; Dworkin, 1981) and adaptation (Sen, 1985). Famously, Sen (1985, p. 21) argued that: “A person who is ill-fed, undernourished, unsheltered and ill can still be high up in the scale of happiness or desire-fulfillment if he or she has learned to have ‘realistic’ desires and to take pleasure in small mercies.” As a result, subjective measures raise some normative concerns when used to identify the worst-off in theories of justice and public policy (Adler, 2012; Decancq et al., 2015b; Nussbaum, 2008). Third, the preference-based approach has its roots in micro-economic consumer theory (Deaton & Muellbauer, 1980) and uses individual preferences—that is, the opinions of the individuals concerned on the relative importance of different aspects of well-being—combining these with explicit fairness principles to construct an interpersonally comparable measure of well-being (Fleurbaey & Maniquet, 2011). The preference-based approach raises new measurement challenges, as it requires information on the opinions of the individuals concerned on the relative importance of the different well-being dimensions.

Gerontologists have long and forcefully criticized the paternalistic nature of an objective approach in which experts or researchers define the meaning of well-being at an older age (Phelan et al., 2004; Whitley et al., 2020). According to Bowling and Dieppe (2005, pp. 1548–1550), for instance, there is “little point in developing policy goals if elderly people do not regard them as relevant.” Explicit critique of the subjective approach to constructing a measure for interpersonal well-being comparisons at an older age is more recent in the gerontological literature (Decancq & Michiels, 2019). However, the longstanding literature on psychological processes and person–environment transactions to compensate for age-related decline (see, e.g., Baltes & Baltes, 1990; Carstensen et al., 2003; Lawton, 1985) provides ample evidence that Sen’s critique of the subjective approach is not a mere philosophical note in the margin. As a result, there is growing interest in preference-based measures for well-being comparisons at an older age (Makai et al., 2014) whereby life domain scores are converted into a summary well-being score using older people’s opinions about the relative importance of the life domains (see, e.g., Coast et al., 2008).

Although there is a long tradition of including lay views in the definition of well-being at older age, few studies have investigated older people’s own views on the importance of well-being domains (Molzahn et al., 2010). In these studies, older people are asked to rate or rank well-being domains according to their importance. Although they have been conducted in different countries, the results of previous studies using rating scales are remarkably consistent and show that social relationships, health and autonomy are the most highly rated well-being dimensions (see, e.g., Beaumont & Kenealy, 2004; Charbonneau-Lyons et al., 2002; Henchoz et al., 2015; Hsu, 2007; Kalfoss & Halvorsud, 2009; Molzahen et al., 2010; Phelan et al., 2004; Wilhemson et al., 2005; Zhang et al., 2018). Using a ranking exercise, Hsieh (2005) observed that health is the most important well-being domain among older people in the United States, followed by family life, religion, friendships, financial situation, leisure time, neighborhood, and work. Although these studies offer valuable information on lay conceptions of well-being, they do not provide information about the intensity of the relative importance of the well-being dimensions. Knowing that the dimension of “health” is rated as extremely important and the dimension of “financial situation” as very important, for instance, does not provide us with sufficiently detailed information to compare the well-being of an older person who is in slightly better health with an older person who is in a much better financial situation.

The current article presents the factorial survey as an innovative approach to obtaining information about the opinions of older persons on the relative importance of the different aspects of well-being. The factorial survey is an experimental method in which respondents are asked to rate several hypothetical descriptions of objects or situations (called vignettes; Auspurg & Hinz, 2015; see Author Note 1). This approach provides detailed information about the relative importance of the different dimensions and is, to the best of our knowledge, novel in the context of research on well-being at an older age. Although the factorial survey has been applied in a wide range of academic disciplines to address human judgments (for an overview, see Wallander, 2009), we are aware of only one other study that used it for the related purpose of measuring successful aging (Whitley et al., 2020). Conducting a factorial survey among a general population sample, Whitley et al. (2020) found that cognitive functioning and disability are the most important dimensions of successful aging, while disease and productive engaging are the least. Because of their multifactorial design and sometimes complex vignette descriptions, factorial surveys may put greater cognitive burden on respondents compared with standard survey questions. We therefore investigated the temporal reliability of our findings and evaluated response time and response consistency.

We illustrate the potential of the factorial survey method with data from a repeated survey experiment administered by the survey agency Qualtrics. Given that respondents were interviewed five times between May and December 2020, the data contain unique longitudinal information with which we assess the reliability of the factorial survey (see Author Note 2). The data were collected among respondents aged 50 years or older in Flanders, the Dutch-speaking northern part of Belgium. Like many Western European countries, Belgium is facing significant challenges due to population aging. Life expectancy was 82.1 years (in 2019), with a high number of people leaving the workforce before the official retirement age of 65 through early retirement schemes. Belgium is known for having a Bismarckian comprehensive social security system, with compulsory health insurance that covers nearly the entire population (Gerkens & Merkur, 2020; Marx & Van Cant, 2019).

The remainder of this article is structured as follows. The second section discusses the research methodology, including the design of the factorial survey, the data collection, the survey sample, and the analysis techniques. The third section presents the results. The final section presents our conclusions and discusses the study’s main limitations and implications for policy, practice, and research.

## Research Design and Methods

### Design of the Factorial Survey

The factorial survey is an experimental method that presents respondents with several hypothetical descriptions of an object or situation to assess how people make judgments across multidimensional phenomena (Auspurg & Hinz, 2015). We used a factorial survey to explore the judgments of older people about well-being. Central to this approach is the use of vignettes. A vignette typically contains a combination of randomly selected values (levels) from different dimensions that are assumed to be relevant to the judgment being studied (Auspurg & Hinz, 2015; Rossi & Anderson, 1982). The respondents’ task is to express their evaluation of each vignette on a rating scale. During this process, respondents might take multiple dimensions into account and give more weight to the outcomes in some dimensions than others. Using multivariate analysis techniques, researchers can then examine the impact or the relative importance of each level of a dimension on the variation in vignette ratings (Jasso, 2006).

A crucial step in the design of any factorial survey is the selection of dimensions and levels within the vignettes. We used an extensive literature review to select the well-being dimensions. More precisely, we included the dimensions of “health,” “social relations,” “income,” “leisure,” “engagement,” and “religion,” as qualitative studies suggest that these are of major importance in lay views of well-being (see, e.g., Brown et al., 2004; Hung et al., 2010; Van Leeuwen et al., 2019). For each dimension, we specified four dimension levels. An overview of the dimensions and levels can be found in Table 1.

**Table 1.** Vignette Dimensions and Levels

Dimension	Description	Level
Health	Physical or mental health problems	Severe/moderately severe/nonsevere/no
Social relations	Contact with family or friends	No/less than once per week/once per week/several times per week
Income	Total net household income per month	€1,500.00/€2,700.00/€3,900.00/ €5,000.00
Leisure	Hobby or leisure activities	No/less than once per week/once per week/several times per week
Engagement	Useful or meaningful activities	No/less than once per week/once per week/several times per week
Religion	Time spent on religion or spirituality	No/less than once per week/once per week/several times per week

*Notes*: The income cutoffs were derived from the quintile values of the income distribution of people aged 50 years or older in Belgium (Eurostat, 2020).

**Figure 1.**
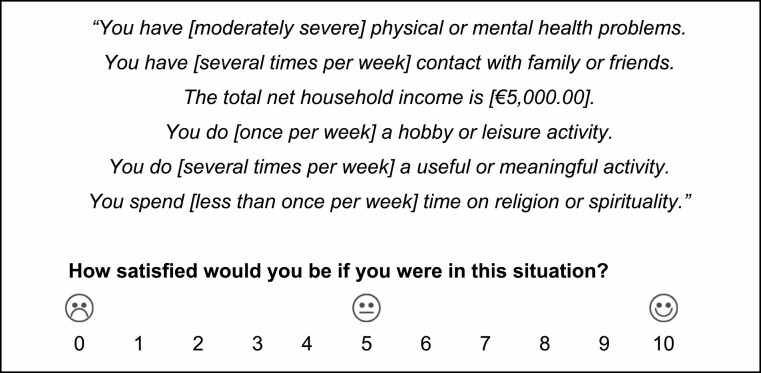
Example vignette. The words between brackets are the levels that varied experimentally from vignette to vignette. The order of the dimensions within the vignettes varied across the vignette sets to avoid potential order effects.

To familiarize respondents with the dimensions and levels, we first asked them to indicate their own performance on each of the vignette dimensions. Afterwards, participants were presented with the vignettes and asked to indicate, on an 11-point satisfaction scale, how much well-being each hypothetical life situation would bring about according to them. An example can be found in Figure 1.

In a factorial survey with six dimensions and four levels, there are 4,096 possible combinations of dimension levels, which constitute the vignette population. As it is undesirable to completely administer the entire vignette population in the survey (see, e.g., Sauer et al., 2011, on cognitive overload), researchers usually draw a smaller subset of vignettes (Atzmüller & Steiner, 2010). We selected 50 different subsets of seven vignettes each, using a computer algorithm (provided by the SAS macro %Mktex) to create a D-efficient design. This approach ensures that all effects of the vignette dimensions can be estimated with the maximum amount of statistical precision. It looks, in particular, for a sample which is as close as possible to being balanced (i.e., the levels of each dimension occur equally) and orthogonal (i.e., equal occurrence of each possible combination of levels; for more details, see Dülmer, 2007). The final D-efficiency value of our sample was 99.99%, indicating an almost perfectly balanced and orthogonal design. We randomly assigned respondents to one of the vignette sets.

### Data Collection and Survey Sample

We implemented the factorial survey in a repeated survey experiment among people aged 50 years or older in Flanders, the Dutch-speaking northern part of Belgium. Respondents were followed at five different time points between May and December 2020. There was an interval of 1 month between each follow-up survey, with the exception of the last, which took place 10 weeks after Wave 4 (see Table 2 for an overview).

**Table 2.** Overview Waves

Wave	Period	Sample size	
		Total	Recontacts
Baseline	7–13 May	800	—
Wave 2	10–21 June	781	452
Wave 3	22 July–4 August	827	298
Wave 4	4–18 September	762	215
Wave 5	1–17 December	764	154

We recruited participants from an online panel administered by Qualtrics, a survey agency that employs nonprobability sampling strategies in developing its sample frame. A total of 1,003 respondents participated in the baseline survey (i.e., Wave 1), for which we set cross-quotas on age and gender to obtain a balanced sample. We removed all cases in which respondents had obviously been distracted while answering the factorial survey or paused the survey and returned to it at a later point in time. This meant that we discarded interviews that took longer than 24 hr to complete, as well as observations that deviated by twice the standard deviation from the mean reaction time for a single vignette (for the recommendation of this procedure, see Mayerl & Urban, 2008).

As a result, the final sample for the baseline interview comprised 800 respondents. Of these respondents, 19.25% (*n* = 154) also participated in the four follow-up surveys (i.e., Waves 2–5), in which the samples were drawn on a natural fallout basis (i.e., without demographic quotas). More specifically, we first sent invitations to respondents who had previously participated and only when this pool of recontacted respondents was exhausted did we approach new panel members. Respondents received an incentive from Qualtrics (either a flat fee or a points system) based on the length of the survey, their specific profile and target acquisition difficulty.

A drop-out analysis showed no significant differences between respondents who participated in all five waves and those who did not (see Supplementary Table 1). We therefore only provide an overview of the sample characteristics from the baseline survey in the first wave (see Table 3). The average age in the sample was 64.66 (*SD* = 7.61). Consistent with the official retirement age, the majority of the participants were retired. Approximately half were male, half received higher education, and almost half had equivalized disposable incomes of €2,000 or more. The representation of lower-educated respondents and older adults with a migration background was low. Around one third of respondents reported having long-term health problems and another third felt limited in daily activities due to their health.

**Table 3.** Sample Characteristics From the Baseline Survey (Wave 1)

Characteristic	%
Male	53.25
Age	
50–64 years	47.75
65–74 years	41.38
75 years or more	10.87
Highest educational degree	
No or primary	5.12
Secondary	43.38
Higher	51.50
Retired	63.75
Eq. disposable household income	
<€1,500.00	19.25
€1,500.00–€1,999.99	36.38
€2,000.00–€2,999.99	29.50
≥€3,000.00	14.87
Migration background	7.88
Having long-term health problems	33.87
Being disabled	30.13
Observations	800

### Analysis

In the first part of the analysis, we explored the relative importance of the well-being dimensions. A factorial survey produces multilevel data, as vignettes are nested within respondents (Hox et al., 1991). Therefore, we estimated a multilevel random intercept model with vignettes as the Level 1 unit of analysis and respondents as Level 2. The estimated model can be specified as follows:


$$\begin{array}{l} S_{ij}=\alpha_{0}+\beta x_{ij}+\gamma_{ij}z_{ij}+u_{0j}+e_{0ij} \end{array}$$

where *S*_*ij*_ represents the satisfaction score given by respondent *i* to vignette *j*; $x_{ij}$ is a vector of variables related to the vignette dimension levels (with the lowest level as the reference case); $z_{ij}$ is a vector of control variables for the vignette position and vignette set; $u_{0j}$ is the error component on Level 2 capturing the between-respondents variation; and $e_{0ij}$ is the Level 1 error component measuring the variation within respondents. We assumed that both error terms are independently and normally distributed with zero means and constant variances (Hox et al., 1991). To check heterogeneity of the relative importance of the well-being dimensions, we repeated the analysis by gender and age groups (i.e., 50–64, 65–74, and 75 or older). Moreover, as a sensitivity check, we repeated all models including control variables for respondents’ actual circumstances in each of the well-being domains (see Model 2 in Supplementary Table 2).

The estimated β coefficients in the model capture the relative importance of the different well-being dimensions. They measure the net increase or decrease in the satisfaction score of the vignette of a particular dimension level compared to the lowest dimension level (i.e., reference category; Jasso, 2006). When comparing the coefficients within and between dimensions, the size of the changes in the levels (see Table 1) needs to be taken into account (Hauber et al., 2016). The coefficient of the second level in the income dimension, for instance, captures the increase in the satisfaction score of an income increase from “€1,500” to “€2,700,” whereas the coefficient of the second level in the health dimension captures the increase in the satisfaction score of an increase in health from having “severe health problems” to “moderately severe health problems.”

Next, we examined the temporal reliability of the factorial survey by comparing the estimated coefficients between a test and retest period. To do this, we first pooled the data of the test and retest periods (e.g., Waves 1 and 2). We then interacted the coefficients with a dummy that equaled 0 for the test and 1 for the retest period. Differences in the results of the two time points were formally tested using a Wald test of joint significance of the interaction terms. If the test revealed that there were no significant differences in the coefficients, it would imply that the results of the factorial survey were temporally reliable. In total, we performed 10 different test–retest analyses comparing each possible pair of waves. In order to have a stable sample size across these different test–retests, we only included respondents who participated in all five waves. We then repeated the analysis using the largest possible sample for each pair of waves as a sensitivity check (see Supplementary Table 3).

Finally, we computed two indicators of cognitive load: response time and response consistency. Response time was available for every single vignette and measured in seconds. Following Sauer et al. (2011), response consistency was obtained from the unexplained variance in the vignette evaluations of each respondent. More specifically, we took the square of the Level 1 residuals from the multilevel random intercept model described above. The higher the consistency of responses, the smaller the amount of variance that was unexplained by the vignette dimensions. Low residual values (approaching zero) thus reflected high levels of response consistency, whereas high residuals indicated low consistency in responses (Sauer et al., 2011).

For both response time and response consistency, we analyzed whether they were systematically related to respondent characteristics and whether they changed across the course of the vignette evaluations. This latter analysis provided insights into the presence of potential learning or fatigue effects across the sequence of vignette evaluations. We took the following respondent characteristics into account: age (i.e., 50–64, 65–74, and 75 or older), education (i.e., none or primary, secondary, and higher education), immigration background, equivalized disposable income (i.e., <€1,500.00; €1,500.00–€1,999.99; €2,000.00–€2,999.99; and ≥€3,000.00), being retired, suffering from long-term health problems, and being disabled. We measured income at the household level. The equivalized income was obtained by dividing the household income by the square root of the household size. We operationalized migration background by country of birth. A person was classified as having a migration background if they or at least one of their parents were born abroad. We assessed suffering from a chronic disease using a question on whether respondents suffered from physical or mental health problems that had lasted (or were expected to last) 6 months or more. We defined being disabled as facing limitations in daily activities due to physical or mental health problems.

We estimated all models within STATA 16 using the generalized least squares (GLS) estimator with cluster-robust standard errors.

## Results

### Relative Importance of Well-Being Dimensions

**Figure 2.**
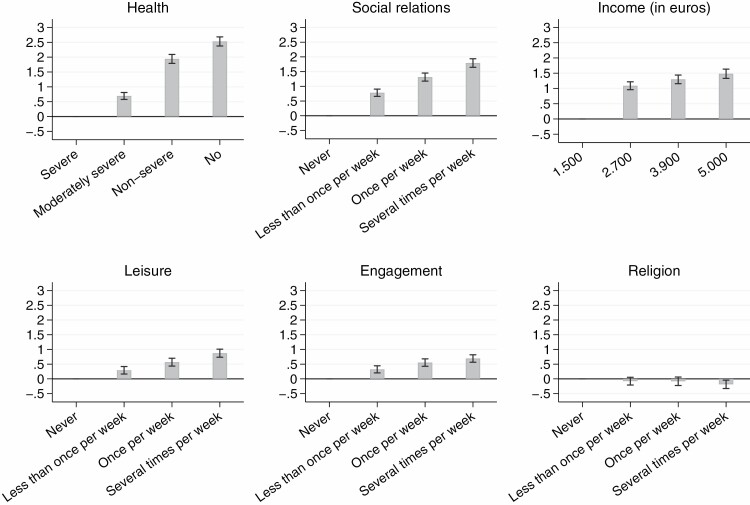
Visualization of the relative importance of the dimension levels based on the estimated coefficients (and 95% confidence interval; *n* = 800). Based on a multilevel regression (generalized least squares) with robust standard errors. The coefficients were estimated including controls for design effects (i.e., vignette position and dummy variables for vignette set). The regression coefficients along with the standard errors can be found in Supplementary Table 2.

With the exception of religion, all estimated coefficients were significantly different from zero and pointed in the anticipated direction. Indeed, Figure 2 shows that, within each dimension, respondents attributed higher weights to higher levels. The estimated coefficients for the income dimension indicate, for instance, that having an income level of “€2,700” has a significantly positive effect of 1.09 points (on the 11-point satisfaction scale) compared with the reference category of “€1,500,” but that the additional gain of having higher income levels is small. The effect of improving the health dimension from “moderately severe problems” to “non-severe problems” was substantially higher compared with the effect of an improvement from “severe problems” to “moderately severe problems” and from “non-severe problems” to “no health problems.”

The magnitude of the estimated coefficients not only varied within dimensions, but also between dimensions. A change from “severe health problems” to “no health problems” would, according to our participants, increase their well-being by 2.53 points, while a similar change (i.e., from the lowest to the highest level) in social relations and income would result in a well-being increase of 1.79 and 1.48 points, respectively. A change in leisure and engagement activities from “never” (i.e., the lowest level) to “several times a week” (i.e., the highest level) would result in an increase of only 0.87 and 0.69 points, respectively. Investing time in religion appeared to generate little well-being according to our respondents, except when the frequency increases to “several times per week.” At that level, the effect of religion becomes significant and the vignette rating drops by 0.19 points. These results were consistent across age groups and gender, although male respondents gave more importance to having a high income (€5,000 per month) compared to female respondents (see Supplementary Figure 1). The heterogeneity between the younger and older age groups was not statistically significant (see Supplementary Figure 2).

One important finding was that the ranking of the well-being dimensions according to their relative importance depends on the change considered in the dimension levels. Figure 2 shows that respondents preferred an increase in the income dimension from the lowest to the second level over an increase in the health dimension from the lowest to the second level. This is, however, no longer the case when increases from the lowest to the third or fourth levels were considered. In these cases, respondents preferred a change in the health dimension over an increase in the income dimension.

### Temporal Reliability

Thus far, we have explored the relative importance of the different well-being dimensions based on the estimated coefficients. In order to examine whether these coefficients were reliable, we estimated a pooled model with additional interaction terms between the coefficients and a dummy for the retest period. Subsequently, a Wald test of joint significance of the interactions was used to evaluate whether the results from the test and retest diverged (see Table 4).

**Table 4.** Wald Test of Equal Coefficients Between Test and Retest (*n* = 154)

Retest	Test			
	Wave 1	Wave 2	Wave 3	Wave 4
Wave 2	χ ^2^ = 37.48**			
Wave 3	χ ^2^ = 38.32**	χ ^2^ = 24.31^ns^		
Wave 4	χ ^2^ = 31.68*	χ ^2^ = 20.01^ns^	χ ^2^ = 20.69^ns^	
Wave 5	χ ^2^ = 22.21^ns^	χ ^2^ = 22.50^ns^	χ ^2^ = 18.73^ns^	χ ^2^ = 29.14*

*Notes*: Only respondents who participated in all five waves were included in the analysis (*n* = 154).

df = 18; ^ns^*p* > .05, * *p* < .05, ** *p* < .01, *** *p* < .001.

In 6 of 10 cases, the null hypothesis of similar parameter estimates could not be rejected at the 5% significance level—providing support for the temporal reliability of the estimated coefficients. However, the relative importance of the well-being dimensions in Wave 1 proved to be statistically different from the relative importance of the dimensions in Waves 2, 3, and 4. In addition, we found that the estimated coefficients changed significantly between Wave 4 and Wave 5. The most noticeable difference over time was related to the income dimension. More precisely, we found that income was perceived to be less important in Wave 1 than in the consecutive waves (results not reported here).

### Feasibility

Response behavior provides valuable information on the cognitive load of the factorial survey and allows us to detect potential problems in handling the vignette evaluations. Next, we take a closer look at two indicators of response behavior: response time and response consistency.

#### Response Time

Overall, respondents needed 2.55 min to complete the entire vignette module. This is equivalent to an average of 21.83 seconds per vignette. Model 1 in Table 5 displays the effects of respondent characteristics on response time per vignette. The results suggested that older respondents required more time than younger respondents. More precisely, it took the oldest participants (i.e., 75 years or older) almost 5 s longer to rate a single vignette compared with the youngest respondents (i.e., 50–64 years). Likewise, the average response time per vignette was longer among respondents aged 65–74 years than among those aged 50–64 years. Moreover, we found that women and highly educated respondents took more time to rate the vignettes, compared with men and lower-educated respondents. However, in contrast to the effect of age, these findings were not robust across waves (see Supplementary Table 4).

**Table 5.** Random Intercept Models of Response Time (RT) and Response Consistency (RC)

Variable	Model 1 RT		Model 2 RC	
	*b*	SE	*b*	SE
Male (1 = yes)	−1.50*	(0.67)	0.17	(0.16)
Age (ref. 50–64 years)				
65–74 years	2.01*	(0.83)	−0.16	(0.23)
75 years and older	4.91***	(1.29)	0.17	(0.36)
Education (ref. no or primary)				
Secondary	2.14	(1.10)	0.10	(0.37)
Higher	2.47*	(1.09)	−0.16	(0.36)
Retired (1 = yes)	0.18	(0.88)	0.04	(0.24)
Eq. disposable household income (ref. < €1,500.00)				
€1,500.00–€1,999.99	−0.51	(0.83)	−0.07	(0.23)
€2,000.00–€2,999.99	0.23	(0.97)	−0.50*	(0.23)
≥€3,000.00	−1.47	(1.14)	−0.62*	(0.24)
Migration background (1 = yes)	−1.94	(1.15)	0.50	(0.39)
Having long-term health problems (1 = yes)	−0.70	(0.93)	0.57*	(0.23)
Being disabled (1 = yes)	1.88	(0.98)	0.10	(0.24)
Baseline speed	0.00	(0.00)		
Constant	24.27***	(1.83)	2.06***	(0.60)
Sigma_u	7.49		1.55	
Sigma_e	12.76		3.73	
Wald chi²	811.00		117.01	
*p*-Value	.000		.000	
*R*²	11.6%		3.3%	
Respondents	800		800	
Vignettes	5,600		5,600	

*Notes*: Based on a multilevel regression (GLS) with robust standard errors. Tested with controls for design effects (i.e., vignette position and dummy variables for vignette set). Baseline speed is defined as the time that a person needs to answer questions, independent of the content. It was measured by subtracting the response time of the vignette module from the entire survey length.

* *p* < 0.05, ** *p* < 0.01, *** *p* < 0.001.

**Figure 3.**
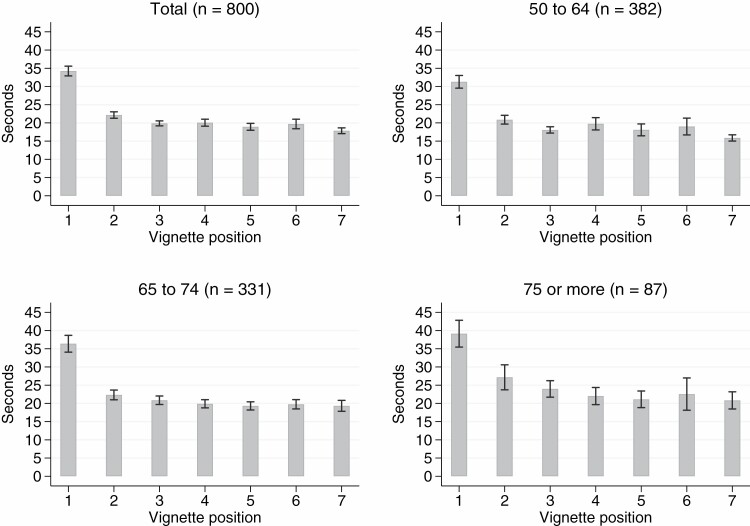
Average response time (and 95% confidence intervals) by vignette position.

Figure 3 shows the evolution of response time across the sequence of vignettes. Given that older participants needed more time to evaluate a vignette, the age variable warrants special consideration. In general, we observed that the response speed was lowest at the beginning of the vignette module. Afterwards, the response time decreased rapidly and stabilized after the third vignette. Across the entire sequence of vignettes, the response speed of older respondents was generally lower than the response speed of younger respondents. However, we observed the same pattern, that is, a sharp decline in response time within the first part of the vignette module, in all age groups.

#### Response Consistency

Model 2 in Table 5 predicts the absolute value of the squared residual, which was used as an indicator of response consistency by respondent characteristics. A positive coefficient reflects an increase in the inconsistency of the vignette ratings. Although older age groups showed a longer response time, there was no indication that their responses were less consistent. Significant effects did emerge, however, for income and the variable related to suffering from a chronic disease. More specifically, those with a higher income (i.e., between €2,000.00 and €2,999.99 or ≥€3,000.00) were more consistent in evaluating the vignettes than individuals with a low income (i.e., <€1,500.00). The responses of participants with long-term mental or physical health problems, on the other hand, were less consistent than those of the respondents without mental or physical health problems. The latter effect was not confirmed in other waves (see Supplementary Table 5).

**Figure 4.**
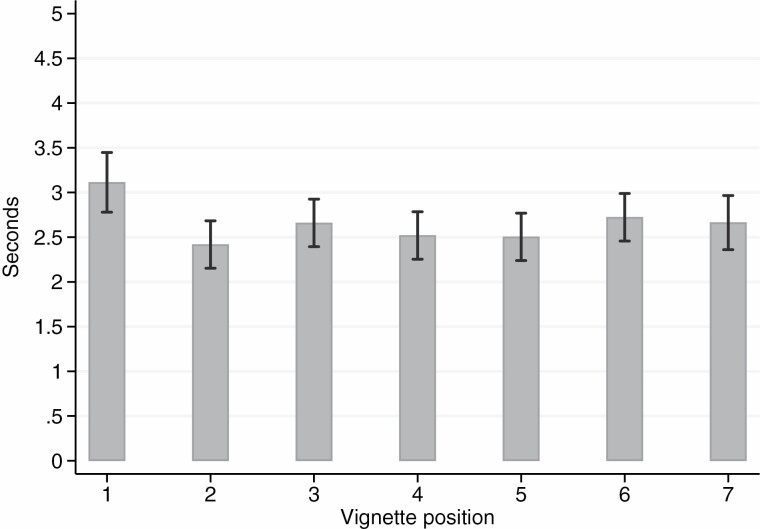
Average response consistency (and 95% confidence intervals) by vignette position (*n* = 800).

As can be seen from Figure 4, the level of response inconsistency drops significantly after the first vignette but remains relatively stable afterwards. After the first vignette, respondents are thus able to make efficient judgments at a stable level of response consistency. We observed this pattern among all respondents—including the oldest, lowest-educated, and those with physical or mental impairments (not reported here). These results hint in the direction of learning effects.

## Discussion and Implications

In this study, we investigated the potential of a factorial survey to estimate the relative importance of well-being dimensions among older people. Overall, the results confirmed earlier findings that, according to the older population, well-being is multidimensional (Brown et al., 2004; Hung et al., 2010; Phelan et al., 2004). Indeed, our results suggest that health, social relations, financial resources, leisure time, and active engagement are all important to the well-being of older people. Not all dimensions are, however, equally important. A change from “severe health problems” to “no health problems” had the highest relative importance, followed by a change in social relations from “never” to “several times a week” and a change in income from “€1,500” to more than “€5,000.” These results are in line with other studies, which found health to be the most important dimension, followed by social contacts (Beaumont & Kenealy, 2004; Charbonneau-Lyons et al., 2002; Hsieh, 2005; Hsu, 2007; Phelan et al., 2004; Whitley et al., 2020; Zhang et al., 2018) and income (Chen & Olsen, 2022). Changes in leisure time and engaging activities, by contrast, played a smaller role. Religion appeared to be unimportant for the respondents. This last result stands in contrast with a study conducted by Hsieh (2005), who found that, according to older adults in the United States, religion was the third most important well-being domain among eight well-being domains studied. One plausible explanation for this may be found in the process of secularization that has marked Western European societies, including Flanders, since the 1960s (Dobbelaere, 2002).

We illustrated, moreover, that the relative importance of the well-being dimensions depends on the size of the changes in the dimension levels. In line with the economic literature on the diminishing marginal utility of income (see, e.g., Layard et al., 2008), we found that respondents clearly disliked being poor (i.e., the lowest level), but the additional gains of higher-income levels were relatively small. Likewise, moving from “moderately severe” (i.e., the second level) to “non-severe” health problems (i.e., the third level) resulted in much higher gains in older people’s well-being than changes in the other health levels.

The estimated coefficients of the relative importance of the well-being dimensions proved to be reliable in 6 out of 10 test–retest analyses performed. In fact, the level of consistency over time between the tests and retests was quite remarkable considering that respondents evaluated a different set of vignettes each time. In the other four cases, the results of the tests and retests were slightly different, mainly because the attributed importance of income was lower in Wave 1 compared with the other waves. Further research is needed to interpret this result conclusively. However, due to the coronavirus disease 2019 (COVID-19) pandemic and subsequent policy measures to control the spread of the coronavirus, it is likely that people have adapted their life goals to reflect changing life circumstances (for a discussion, see also Bland, 2020). Given that a large part of our sample was retired, and pensions remained stable, income was one of the few well-being dimensions that did not dramatically change during the COVID-19 crisis for the respondents. Perhaps this may explain why the respondents perceived income as less important in Wave 1.

The analyses of response time and response consistency provided further evidence that the respondents coped well with the complexity of the factorial survey. Overall, variation in response time between respondents with different characteristics was small, although there was a tendency to lower response speed in the older age groups. According to Auspurg et al. (2009) and Sauer et al. (2011), such an age effect is inherent to any question type and therefore not indicative of problems specific to a factorial survey. In line with a previous feasibility study (Teti et al., 2016), respondents from different age groups and educational backgrounds showed similar levels of response consistency. Regarding household income, we did find that the inconsistency in responses was higher among low-income earners, again confirming the results of Teti et al. (2016).

Finally, we found no signs of cognitive overload across the sequence of vignette evaluations. On the contrary, the results pointed more in the direction of learning effects. For both response time and response inconsistency, a substantive drop was observed after the first vignette: apparently, respondents needed some time to become familiar with the rating task at hand. Nevertheless, the first vignette evaluations were already acceptable in terms of response time and consistency. Two remarks need to be made in this respect. First, it is important to emphasize that we asked respondents at the beginning of the survey to indicate their own level in each vignette dimension and to rate a vignette of their own life. Respondents were thus already familiar with the vignette descriptions before the actual factorial survey experiment started. Second, we presented only seven vignettes to the respondents. It could thus be true that cognitive overstrain may occur if respondents have to evaluate a higher number of vignettes (Auspurg et al., 2009; Sauer at al., 2011; Teti et al., 2016).

### Limitations and Future Research

The results of this study should be interpreted in light of several limitations. First of all, it is possible that the relative importance given to the different well-being dimensions was influenced by the way they were operationalized. Research suggests, for instance, that the impact of the social network on subjective well-being in later life depends on the quality of interpersonal relationships rather than on the network size and the contact frequency (Bruine de Bruin et al., 2020; Carstensen et al., 2003; Pinquart & Sörensen, 2000). Focusing on qualitative rather than on quantitative indicators of social relations could yield different results. Additional research is thus needed to test the sensitivity of our results to the specific operationalization of the well-being dimensions.

Second, we conducted this study in an online setting, in which respondents were drawn from a nonprobability panel. As certain subpopulations may self-select into such panels, the generalizability of our results to the general population of older people might potentially be affected. In fact, our sample was predominantly White, highly educated, and in good health. Moreover, it is probable that the Qualtrics panel consists mainly of experienced survey participants, who are likely to be more familiar with cognitively demanding survey questions than inexperienced individuals would be. Future research is needed to investigate whether our findings can be replicated among more heterogeneous and representative samples of the older population.

As the example of religion suggests, the relative importance of the well-being dimensions might be influenced by factors such as the surrounding culture. An interesting avenue for future research would be to repeat this factorial survey experiment in other populations and different geographic regions. Indeed, an important point that needs to be noted here is the specific research context of this study. The research took place during the COVID-19 pandemic. A closer examination is warranted to identify how institutional and contextual characteristics might have impacted the results.

Finally, some preliminary efforts were made to understand how the relative importance of the well-being dimensions vary according to respondent characteristics such as age and gender. More in-depth exploration is needed, however, to fully capture potential inter- and intracultural variations in what matters to older people. In addition, future work could investigate how current life circumstances and experiences across the life course shape older people’s views on what matters in life and, hence, influence the relative importance given to different well-being dimensions.

### Implications

Despite its limitations, this study provides ample evidence that the factorial survey method can be used to derive information about the relative importance of different well-being dimensions and, hence, to draw a tailored picture of older people’s views on well-being. This finding has several implications for policy, practice, and research.

First, it encourages policymakers and practitioners to use the factorial survey method for the development of preference-based interventions and person-centered care. The factorial survey is a useful tool to identify priorities in aging policies—especially when choices between different well-being dimensions are involved. For instance, when resources are scarce, policymakers may want to prioritize interventions on the basis of the importance given by older people themselves. In addition, empirical evidence based on the view of the population concerned may provide guidance in decision-making when policies have an opposite impact on different dimensions of well-being (social distancing measures, for instance, reduce the risk of being infected with COVID-19, but lead to isolation and reduced social contacts).

Finally, this study contributes to the awareness that, according to older people, not all well-being dimensions are equally important. However, the assumption of equal importance between well-being dimensions is often made in the literature and implicitly embedded in many well-being measures (Decancq & Lugo, 2013). The results of this study open the door to a more nuanced approach, in which well-being dimensions are weighted according to their relative importance using a factorial survey. Moreover, existing well-being measures in the gerontological literature often fail to take into account inter- and intracultural variation in preferences about the relative importance of well-being dimensions. However, several studies have shown that older persons themselves use different criteria to evaluate well-being domains and place different emphasis on their importance to overall well-being (Hsieh, 2005; Hung et al., 2010; Whitley et al., 2020; Wilhelmson et al., 2005). Exploring the potential of the factorial survey to elicit individual or subgroup-level information on the relative importance of the well-being domains would be an important next step in developing a desirable yardstick to compare the well-being of older people.

## Conclusion

Due to the rapid aging of our society, the need to evaluate health and social care services for older people is expected to grow considerably. An accurate measurement of well-being, including the weighing of well-being dimensions, is indispensable in this regard, and choosing the appropriate methodology to do so has become all the more relevant (Himmler et al., 2021). Against this background, this study investigated the potential of a factorial survey to derive the relative importance of well-being dimensions among older people. Overall, the estimated importance of the dimensions proved to be plausible and reliable. In addition, we observed a high level of response consistency within an acceptable amount of response time. We believe, therefore, that factorial surveys offer us a promising way forward in eliciting the views of older people on well-being, and, hence, in developing policies that matter to them.

## Supplementary Material

igac034_suppl_Supplementary_Material

## Author Notes

1. Other, but similar, survey-based experiments are discrete choice experiments (see, e.g., Chen & Olsen, 2022) and best–worst scaling exercises (see, e.g., Coast et al., 2008).

2. The survey took place during the COVID-19 pandemic. Affecting the older population in particular, the first wave in Belgium started in March 2020 and declined significantly toward summer. In August 2020, the number of daily cases increased again—this time particularly affecting the younger age groups. Following a peak in August, COVID-19 incidence fell slightly, before two successive phases of rapid incline, in the first half of September and again in October 2020.

## References

[CIT0001] Adler, M. D . (2012). Happiness surveys and public policy: What’s the use? Duke Law Journal, 62(8), 1509–1601. https://scholarship.law.duke.edu/dlj/vol62/iss8/2

[CIT0002] Adler, M. D., & Fleurbaey, M.(Eds.). (2016). The Oxford handbook of well-being and public policy. Oxford University Press.

[CIT0003] Adler, M. D., & Norheim, O. F.(Eds.). (2022). Prioritarianism in practice. Cambridge University Press.

[CIT0004] Arrow, K. J . (1973). Some ordinalist-utilitarian notes on Rawls’s theory of justice by John Rawls. The Journal of Philosophy, 70(9), 245–263. doi:10.2307/2025006

[CIT0005] Atzmüller, C., & Steiner, P. M. (2010). Experimental vignette studies in survey research. Methodology, 6(3), 128–138. doi:10.1027/1614-2241/a000014

[CIT0006] Auspurg, K., & Hinz, T. (2015). Quantitative applications in the social sciences: Factorial survey experiments. SAGE Publications.

[CIT0007] Auspurg, K., Hinz, T., & Liebig, S. (2009). Complexity, learning effects and plausibility of vignettes in the factorial survey design. Methods, Data, Analyses, 3(1), 59–96. doi:10.12758/mda.2009.003

[CIT0008] Baltes, P. B., & Baltes, M. M. (1990). Psychological perspectives on successful aging: The model of selective optimization with compensation. In P. B. Baltes & M. M. Baltes (Eds.), Successful aging: Perspectives from the behavioral sciences (pp. 1–34). Cambridge University Press. doi:10.1017/CBO9780511665684

[CIT0009] Beaumont, J. G., & Kenealy, P. M. (2004). Quality of life perceptions and social comparisons in healthy old age. Ageing & Society, 24(5), 755–769. doi:10.1017/S0144686X04002399

[CIT0010] Bland, A. M . (2020). Existential givens in the COVID-19 crisis. Journal of Humanistic Psychology, 60(5), 710–724. doi:10.1177/0022167820940186

[CIT0011] Bowling, A., & Dieppe, P. (2005). What is successful ageing and who should define it? BMJ, 331(7531), 1548–1551. doi:10.1136/bmj.331.7531.1548 16373748 PMC1322264

[CIT0012] Brown, J., Bowling, A., & Flynn, T. (2004). Models of quality of life: A taxonomy, overview and systematic review of the literature. Report commissioned by European Forum on Population Ageing Research. University of Sheffield.

[CIT0013] Bruine de Bruin, W., Parker, A. M., & Strough, J. (2020). Age differences in reported social networks and well-being. Psychology and Aging, 35(2), 159–168. doi:10.1037/pag0000415 31697096 PMC7122684

[CIT0014] Carstensen, L. L., Fung, H., & Charles, S. T. (2003). Socioemotional selectivity theory and the regulation of emotion in the second half of life. Motivation and Emotion, 27(2), 103–123. doi:10.1023/A:1024569803230

[CIT0015] Charbonneau-Lyons, D. L., Mosher-Ashley, P. M., & Stanford-Pollock, M. (2002). Opinions of college students and independent-living adults regarding successful aging. Educational Gerontology, 28(10), 823–833. doi:10.1080/03601270290099822

[CIT0016] Chen, G., & Olsen, J. A. (2022). How is your life? Understanding the relative importance of life domains amongst older adults, and their associations with self-perceived COVID-19 impacts. Quality of Life Research. Advanced online publication. doi:10.1007/s11136-021-03043-5 PMC8731135 34988850

[CIT0017] Coast, J., Flynn, T. N., Natarajan, L., Sproston, K., Lewis, J., Louviere, J. J., & Peters, T. J. (2008). Valuing the ICECAP capability index for older people. Social Science & Medicine, 67(5), 874–882. doi:10.1016/j.socscimed.2008.05.015 18572295

[CIT0018] Deaton, A., & Muellbauer, J. (1980). An almost ideal demand system. The American Economic Review, 70(3), 312–326. https://www.aeaweb.org/aer/top20/70.3.312-326.pdf

[CIT0019] Decancq, K., Fleurbaey, M., & Schokkaert, E. (2015a). Inequality, income, and well-being. In A. B. Atkinson & F. Bourguignon (Eds.), Handbook on income distribution, vol. 2A (pp. 67–140). Elsevier.

[CIT0020] Decancq, K., Fleurbaey, M., & Schokkaert, E. (2015b). Happiness, equivalent incomes and respect for individual preferences. Economica, 82, 1082–1106. doi:10.1111/ecca.12152

[CIT0021] Decancq, K., & Lugo, M. A. (2013). Weights in multidimensional indices of wellbeing: An overview. Econometric Reviews, 32(1), 7–34. doi:10.1080/07474938.2012.690641

[CIT0022] Decancq, K., & Michiels, A. (2019). Measuring successful aging with respect for preferences of older people. The Journals of Gerontology, Series B: Psychological Sciences and Social Sciences, 74(2), 364–372. doi:10.1093/geronb/gbx060 28591828

[CIT0023] Dobbelaere, K . (2002). Secularization: An analysis at three levels. Peter Lang. doi:10.3726/978-3-0352-6146-2

[CIT0024] Dolan, P., & Fujiwara, D. (2016). Happiness-based policy analysis. In M. D. Adler & M. Fleurbaey (Eds.), The Oxford handbook of well-being and public policy (pp. 286–317). Oxford University Press.

[CIT0025] Dülmer, H . (2007). Experimental plans in factorial surveys: Random or quota design? Sociological Methods & Research, 35(3), 382–409. doi:10.1177/0049124106292367

[CIT0026] Dworkin, R . (1981). Part 1: Equality of welfare. Philosophy and Public Affairs, 10(3), 185–246. doi:10.4324/9781315199795-6

[CIT0065] Eurostat. (2020). European Union - Statistics on Income and Living Conditions (Version 1) [dataset]. https://ec.europa.eu/eurostat/web/microdata/overview

[CIT0027] Fletcher, G . (2015). Objective list theories. In G. Fletcher (Ed.), The Routledge handbook of philosophy of well-being (pp. 164–176). Routledge.

[CIT0028] Fleurbaey, M., & Blanchet, D. (2013). Beyond GDP: Measuring welfare and assessing sustainability. Oxford University Press.

[CIT0029] Fleurbaey, M., & Maniquet, F. (2011). A theory of fairness and social welfare. Cambridge University Press. doi:10.1017/CBO9780511851971

[CIT0030] Gerkens, S., & Merkur, S. (2020). Belgium: Health system review. *Health Systems in Transition*, 22(5), 1–237. https://apps.who.int/iris/handle/10665/339168 33527904

[CIT0031] Hauber, A. B., González, J. M., Groothuis-Oudshoorn, C. G., Prior, T., Marshall, D. A., Cunningham, C., Ijzerman, M. J., & Bridges, J. F. (2016). Statistical methods for the analysis of discrete choice experiments: A report of the ISPOR conjoint analysis good research practices task force. Value in Health, 19(4), 300–315. doi:10.1016/j.jval.2016.04.004 27325321

[CIT0032] Henchoz, Y., Meylan, L., Goy, R., Guessous, I., Bula, C., Demont, M., Rodondi, N., & Santos-Eggimann, B. (2015). Domains of importance to the quality of life of older people from two Swiss regions. Age and Ageing, 44(6), 979–85. doi:10.1093/ageing/afv130 26404612

[CIT0033] Himmler, S., Soekhai, V., Van Exel, J., & Brouwer, W. (2021). What works better for preference elicitation among older people? Cognitive burden of discrete choice experiment and case 2 best–worst scaling in an online setting. Journal of Choice Modelling, 38, 100265. doi:10.1016/j.jocm.2020.100265

[CIT0034] Hox, J. J., Kreft, I. G. G., & Hermkens, P. L. J. (1991). The analysis of factorial surveys. Sociological Methods & Research, 19(4), 493–510. doi:10.1177/0049124191019004003

[CIT0035] Hsieh, C. M . (2005). Age and relative importance of major life domains. Journal of Aging Studies, 19(4), 503–512. doi:10.1016/j.jaging.2005.07.001

[CIT0036] Hsu, H. C . (2007). Exploring elderly people’s perspectives on successful ageing in Taiwan. Ageing & Society, 27(1), 87–102. doi:10.1017/S0144686X06005137

[CIT0037] Hung, L., Kempen, G., & De Vries, N. (2010). Cross-cultural comparison between academic and lay views of healthy ageing: A literature review. Ageing and Society, 30(8), 1373–1391. doi:10.1017/S0144686X10000589

[CIT0038] Jasso, G . (2006). Factorial survey methods for studying beliefs and judgments. Sociological Methods & Research, 34(3), 334–423. doi:10.1177/0049124105283121

[CIT0039] Kalfoss, M., & Halvorsrud, L. (2009). Important issues to quality of life among Norwegian older adults: An exploratory study. The Open Nursing Journal, 3, 45–55. doi:10.2174/1874434600903010045 19738913 PMC2737120

[CIT0040] Lawton, M. P . (1985). The elderly in context: Perspectives from environmental psychology and gerontology. Environment and Behavior, 17(4), 501–519. doi:10.1177/0013916585174005

[CIT0041] Layard, R . (2005). Happiness: Lessons from a new science. Penguin.

[CIT0042] Layard, R., Mayraz, G., & Nickell, S. (2008). The marginal utility of income. Journal of Public Economics, 92(8–9), 1846–1857. doi:10.1016/j.jpubeco.2008.01.007

[CIT0043] Lee, M. T., Kubzansky, L. D., & VanderWeele, T. J.(Eds.). (2021). Measuring well-being: Interdisciplinary perspectives from the social sciences and the humanities. Oxford University Press. doi:10.1093/oso/9780197512531.001.0001

[CIT0044] Makai, P., Brouwer, W. B., Koopmanschap, M. A., Stolk, E. A., & Nieboer, A. P. (2014). Quality of life instruments for economic evaluations in health and social care for older people: A systematic review. Social Science & Medicine, 102, 83–93. doi:10.1016/j.socscimed.2013.11.050 24565145

[CIT0045] Marx, I., & Van Cant, L. (2019). Belgium’s welfare system: Still lagging after all these years. In S. Blum, J. Kuhlmann, & K. Schubert (Eds.), Routledge handbook of European welfare systems (pp. 38–55). Routledge. doi:10.4324/9780429290510-3

[CIT0046] Mayerl, J., & Urban, D. (2008). Antwortreaktionszeiten in survey-analysen: Messung, auswertung und anwendungen. VS Verlag für Sozialwissenschaften.

[CIT0047] Molzahn, A., Skevington, S. M., Kalfoss, M., & Makaroff, K. S. (2010). The importance of facets of quality of life to older adults: An international investigation. Quality of Life Research, 19(2), 293–298. doi:10.1007/s11136-009-9579-7 20063124

[CIT0048] Nussbaum, M. C . (2008). Who is the happy warrior? Philosophy poses questions to psychology. The Journal of Legal Studies, 37(Suppl 2), 81–113. doi:10.1086/587438

[CIT0049] Phelan, E. A., Anderson, L. A., LaCroix, A. Z., & Larson, E. B. (2004). Older adults’ views of “successful aging”—How do they compare with researchers’ definitions? Journal of the American Geriatrics Society, 52(2), 211–216. doi:10.1111/j.1532-5415.2004.52056.x 14728629

[CIT0050] Pinquart, M., & Sörensen, S. (2000). Influences of socioeconomic status, social network, and competence on subjective well-being in later life: A meta-analysis. Psychology and Aging, 15(2), 187–224. doi:10.1037//0882-7974.15.2.187 10879576

[CIT0051] Pruchno, R. A . (2015). Successful aging: Contentious past, productive future. The Gerontologist, 55(1), 1–4. doi:10.1093/geront/gnv002 26035905 PMC4986598

[CIT0052] Rawls, J . (1982). Social unity and primary goods. In A. Sen & B. Williams (Eds.), Utilitarianism and beyond (pp. 159–186). Cambridge University Press.

[CIT0053] Robbins, L . (1935). An essay on the nature and significance of economic science (2nd ed.). Macmillan.

[CIT0054] Rossi, P. H., & Anderson, A. B. (1982). The factorial survey approach: An introduction. In P. H. Rossi & S. L. Nock (Eds.), Measuring social judgments: The factorial survey approach (pp. 15–67). Sage Publications.

[CIT0055] Ryff, C. D., Boylan, J. M., Kirsch, J. A. (2021). Advancing the science of well-being: A dissenting view of measurement recommendations. In M. T. Lee, L. D. Kubzansky, & T. J. VanderWeele (Eds.), Measuring well-being: Interdisciplinary perspectives from the social sciences and the humanities (pp. 521–535). Oxford University Press.

[CIT0056] Sauer, C., Auspurg, K., Hinz, T., & Liebig, S. (2011). The application of factorial surveys in general population samples: The effects of respondent age and education on response times and response consistency. Survey Research Methods, 5(3), 89–102. doi:10.18148/srm/2011.v5i3.4625

[CIT0057] Sen, A . (1985). Commodities and capabilities. Oxford University Press.

[CIT0058] Teti, A., Gross, C., Knoll, N., & Blüher, S. (2016). Feasibility of the factorial survey method in aging research: Consistency effects among older respondents. Research on Aging, 38(7), 715–41. doi:10.1177/0164027515600767 26282570

[CIT0059] United Nations Department of Economic and Social Affairs, Population Division. (2020). World population ageing 2020 highlights: Living arrangements of older people (ST/ESA/SER.A/451). United Nations

[CIT0060] Van Leeuwen, K. M., Van Loon, M. S., Van Nes, F. A., Bosmans, J. E., De Vet, H., Ket, J., Widdershoven, G., & Ostelo, R. (2019). What does quality of life mean to older adults? A thematic synthesis. PLoS One, 14(3), e0213263. doi:10.1371/journal.pone.0213263 30849098 PMC6407786

[CIT0061] Wallander, L . (2009). 25 years of factorial surveys in sociology: A review. Social Science Research, 38(3), 505–520. doi:10.1016/j.ssresearch.2009.03.004

[CIT0062] Whitley, E., Benzeval, M., & Popham, F. (2020). Population priorities for successful aging: A randomized vignette experiment. The Journals of Gerontology, Series B: Psychological Sciences and Social Sciences, 75(2), 293–302. doi:10.1093/geronb/gby060 29878183 PMC6974399

[CIT0063] Wilhelmson, K., Andersson, C., Waern, M., & Allebeck, P. (2005). Elderly people’s perspectives on quality of life. Ageing & Society, 25(4), 585–600. doi:10.1017/S0144686X05003454

[CIT0064] Zhang, W., Liu, S., & Wu, B. (2018). Defining successful aging: Perceptions from elderly Chinese in Hawai’i. Gerontology and Geriatric Medicine, 4, 25–29. doi:10.1177/2333721418778182 PMC6050632 30035192

