# Supplementary material for: Using a Factorial Survey to Estimate the Relative Importance of Well-Being Dimensions According to Older People: Insights From a Repeated Survey Experiment in Flanders
Source: Innov Aging. 2022 May 19;6(3):igac034. doi: 10.1093/geroni/igac034 (PMC9225818; doi:10.1093/geroni/igac034)
Supplement: igac034_suppl_Supplementary_Material [file igac034_suppl_supplementary_material.docx]

## **Supplementary Table 1.** Logistic regression of dropping out of the survey (n = 800)

| Variable | Odds ratio |  | se |
| --- | --- | --- | --- |
| Male (1 = yes) | 0.88 |  | (0.16) |
| Age (ref. 50 to 64 years) |  |  |  |
| 65 to 74 years | 1.24 |  | (0.30) |
| 75 years and older | 0.97 |  | (0.32) |
| Education (ref. no or primary) |  |  |  |
| Secondary | 1.43 |  | (0.53) |
| Higher | 2.06 |  | (0.78) |
| Retired (1 = yes) | 0.78 |  | (0.20) |
| Eq. disposable household income (ref. < €1,500.00) | | | |
| €1,500.00–€1,999.99 | 1.36 |  | (0.33) |
| €2,000.00–€2,999.99 | 1.31 |  | (0.35) |
| ≥ €3,000.00 | 1.47 |  | (0.48) |
| Migration background (1 = yes) | 1.33 |  | (0.48) |
| Having long-term health problems (1 = yes) | 1.24 |  | (0.33) |
| Being disabled (1 = yes) | 1.06 |  | (0.28) |
| Loglikelihood | -2,698.44 |  |  |
| R² | 1.6% |  |  |
| Respondents | 800 |  |  |
| Vignettes | 5,600 |  |  |

*Note*: Based on a logistic regression with robust standard errors. ^*^ p < 0.05, ^**^ p < 0.01, ^***^ p < 0.001

## **Supplementary Table 2.** The relative importance of the dimension levels

|  | Model 1 | | |  | Model 2 | | |
| --- | --- | --- | --- | --- | --- | --- | --- |
| Variable | b |  | se |  | b |  | se |
| Physical or mental health problems (ref. severe) | | | | | | | |
| Moderately severe | 0.69 | ^***^ | (0.06) |  | 0.68 | ^***^ | (0.06) |
| Non-severe | 1.94 | ^***^ | (0.08) |  | 1.95 | ^***^ | (0.08) |
| No | 2.53 | ^***^ | (0.08) |  | 2.53 | ^***^ | (0.08) |
| Having contact with family or friends (ref. never) | | | | | | | |
| < 1 per week | 0.78 | ^***^ | (0.06) |  | 0.78 | ^***^ | (0.06) |
| 1 per week | 1.31 | ^***^ | (0.07) |  | 1.32 | ^***^ | (0.07) |
| > 1 time per week | 1.79 | ^***^ | (0.07) |  | 1.79 | ^***^ | (0.07) |
| Household income (ref. €1,500.00) | | | | | | | |
| €2.700,00 | 1.09 | ^***^ | (0.07) |  | 1.09 | ^***^ | (0.07) |
| €3.900,00 | 1.30 | ^***^ | (0.07) |  | 1.31 | ^***^ | (0.07) |
| €5.000,00 | 1.48 | ^***^ | (0.08) |  | 1.49 | ^***^ | (0.08) |
| Doing hobbies or leisure activities (ref. never) | | | | | | | |
| < 1 per week | 0.29 | ^***^ | (0.07) |  | 0.28 | ^***^ | (0.07) |
| 1 per week | 0.57 | ^***^ | (0.07) |  | 0.58 | ^***^ | (0.07) |
| > 1 time per week | 0.87 | ^***^ | (0.07) |  | 0.87 | ^***^ | (0.07) |
| Doing useful or meaningful activities (ref. never) | | | | | | | |
| < 1 per week | 0.32 | ^***^ | (0.06) |  | 0.32 | ^***^ | (0.06) |
| 1 per week | 0.55 | ^***^ | (0.06) |  | 0.55 | ^***^ | (0.07) |
| > 1 time per week | 0.69 | ^***^ | (0.06) |  | 0.69 | ^***^ | (0.07) |
| Spending time on religion or spirituality (ref. never) | | | | | | | |
| < 1 per week | -0.08 |  | (0.07) |  | -0.08 |  | (0.07) |
| 1 per week | -0.08 |  | (0.07) |  | -0.08 |  | (0.07) |
| > 1 time per week | -0.19 | ^*^ | (0.07) |  | -0.19 | ^*^ | (0.07) |
| Constant | 0.25 |  | (0.39) |  | 1.56 | ^*^ | (0.74) |
| Controls for actual circumstances on the well-being dimensions |  |  |  |  |  |  |  |
| Sigma_u | 1.28 |  |  |  | 1.22 |  |  |
| Sigma_e | 1.73 |  |  |  | 1.73 |  |  |
| Wald chi² | 2916.54 |  |  |  | 3264.48 |  |  |
| p-value | 0.000 |  |  |  | 0.000 |  |  |
| R² | 32% |  |  |  | 34.9% |  |  |
| Respondents | 800 |  |  |  | 789 |  |  |
| Vignettes | 5600 |  |  |  | 5523 |  |  |

*Note*: Based on a multilevel regression (GLS) with robust standard errors. Model 1 and 2 tested with controls for design effects (i.e., vignette position and dummy variables for vignette set). Model 2 tested with controls for actual circumstances on the well-being dimensions. ^*^ p < 0.05, ^**^ p < 0.01, ^***^ p < 0.001

## **Supplementary Table 3.** Wald test of equal coefficients between test and retest (total sample)

|  |  | Test | | | |
| --- | --- | --- | --- | --- | --- |
|  |  | Wave 1 | Wave 2 | Wave 3 | Wave 4 |
| Retest | Wave 2  (n = 452) | ^ns chi² = 25.57^ |  |  |  |
|  | Wave 3  (n = 298) | ^*** chi² = 56.00^ | ^ns chi² = 20.35^ |  |  |
|  | Wave 4  (n = 215) | ^* chi² = 30.90^ | ^ns chi² = 18.09^ | ^ns chi² =15.20^ |  |
|  | Wave 5  (n = 154) | ^ns chi² = 22.21^ | ^ns chi² = 22.50^ | ^ns chi² = 18.73^ | ^* chi² = 29.14^ |

*Note*: df = 18; ^ns^ p > 0.05, ^*^ p < 0.05, ^**^ p < 0.01, ^***^ p < 0.001

## **Supplementary Table 4.** Random intercept models of response time across waves

|  | Wave 1 | | |  | Wave 2 | | |  | Wave 3 | | |  | Wave 4 | | |  | Wave 5 | | |
| --- | --- | --- | --- | --- | --- | --- | --- | --- | --- | --- | --- | --- | --- | --- | --- | --- | --- | --- | --- |
| Variable | b |  | se |  | B |  | se |  | B |  | se |  | b |  | se |  | b |  | se |
| Male (1 = yes) | -1.50 | ^*^ | (0.67) |  | -2.18 | ^**^ | (0.69) |  | -0.78 |  | (0.72) |  | 0.56 |  | (0.66) |  | 0.18 |  | (0.67) |
| Age (ref. 50 to 64 years) |  |  |  |  |  |  |  |  |  |  |  |  |  |  |  |  |  |  |  |
| 65 to 74 years | 2.01 | ^*^ | (0.83) |  | 2.64 | ^**^ | (0.97) |  | 0.76 |  | (0.10) |  | 0.36 |  | (0.94) |  | 1.66 |  | (0.86) |
| 75 years and older | 4.91 | ^***^ | (1.29) |  | 6.13 | ^***^ | (1.52) |  | 3.45 | ^*^ | (1.505) |  | 2.83 | ^*^ | (1.22) |  | 3.03 | ^**^ | (1.06) |
| Education (ref. no or primary) |  |  |  |  |  |  |  |  |  |  |  |  |  |  |  |  |  |  |  |
| Secondary | 2.14 |  | (1.10) |  | 1.48 |  | (1.40) |  | 0.06 |  | (1.49) |  | 0.04 |  | (1.93) |  | -0.85 |  | (1.75) |
| Higher | 2.47 | ^*^ | (1.09) |  | 0.84 |  | (1.37) |  | 0.20 |  | (1.58) |  | -0.18 |  | (1.96) |  | -0.19 |  | (1.79) |
| Retired (1 = yes) | 0.18 |  | (0.88) |  | 0.29 |  | (1.00) |  | 1.14 |  | (1.11) |  | 1.09 |  | (1.09) |  | -0.75 |  | (0.93) |
| Eq. disposable household income (ref. < €1,500.00) | | | | | |  |  |  |  |  |  |  |  |  |  |  |  |  |  |
| €1,500.00–€1,999.99 | -0.51 |  | (0.83) |  | 1.14 |  | (0.83) |  | 0.31 |  | (0.10) |  | 1.49 |  | (0.97) |  | -0.15 |  | (1.05) |
| €2,000.00–€2,999.99 | 0.23 |  | (0.97) |  | 1.42 |  | (0.96) |  | 0.31 |  | (1.02) |  | 0.66 |  | (1.03) |  | -0.33 |  | (1.07) |
| ≥ €3,000.00 | -1.47 |  | (1.14) |  | 0.12 |  | (1.08) |  | -1.57 |  | (1.18) |  | -0.86 |  | (1.06) |  | -1.80 |  | (1.30) |
| Migration background (1 = yes) | -1.94 |  | (1.15) |  | 0.76 |  | (1.90) |  | -1.87 |  | (1.04) |  | -1.48 |  | (1.77) |  | 0.21 |  | (1.34) |
| Having long-term health problems (1 = yes) | -0.70 |  | (0.93) |  | -1.00 |  | (0.81) |  | -1.47 |  | (0.89) |  | -1.94 | ^**^ | (0.74) |  | 1.11 |  | (0.89) |
| Being disabled (1 = yes) | 1.88 |  | (0.99) |  | 2.41 | ^**^ | (0.91) |  | 2.41 | ^*^ | (0.94) |  | 2.23 | ^**^ | (0.85) |  | 0.71 |  | (0.94) |
| Baseline speed | 0.00 |  | (0.00) |  | 0.00 | ^**^ | (0.00) |  | 0.00 |  | (0.00) |  | 0.00 | ^+^ | (0.00) |  | -0.00 |  | (0.00) |
| Constant | 24.27 | ^***^ | (1.83) |  | 28.41 | ^***^ | (2.78) |  | 27.37 | ^***^ | (2.50) |  | 26.40 | ^***^ | (2.29) |  | 27.18 | ^***^ | (2.78) |
| Sigma_u | 7.49 |  |  |  |  |  |  |  |  |  |  |  |  |  |  |  |  |  |  |
| Sigma_e | 12.76 |  |  |  | 15.68 |  |  |  | 17.56 |  |  |  | 17.19 |  |  |  | 16.60 |  |  |
| Wald chi² | 811.00 |  |  |  | 954.45 |  |  |  | 956.32 |  |  |  | 704.57 |  |  |  | 594.33 |  |  |
| p-value | 0.000 |  |  |  | 0.000 |  |  |  | 0.000 |  |  |  | 0.000 |  |  |  | 0.000 |  |  |
| R² | 11.6% |  |  |  | 12.8% |  |  |  | 9.7% |  |  |  | 7.8% |  |  |  | 8.0% |  |  |
| Respondents | 800 |  |  |  | 781 |  |  |  | 827 |  |  |  | 761 |  |  |  | 763 |  |  |
| Vignettes | 5,600 |  |  |  | 5,467 |  |  |  | 5,789 |  |  |  | 5,327 |  |  |  | 5,341 |  |  |

*Note*: Based on a multilevel regression (GLS) with robust standard errors. Tested with controls for design effects (i.e., vignette position and dummy variables for vignette set). Baseline speed was defined as the general mental speed that a person needs to answer questions, independent of the content of the questions. It was measured by subtracting the response time of the vignette module from the entire survey length.

^*^ p < 0.05, ^**^ p < 0.01, ^***^ p < 0.001

## **Supplementary Table 5.** Random intercept models of response consistency across waves

|  | Wave 1 | | |  | Wave 2 | | |  | Wave 3 | | |  | Wave 4 | | |  | Wave 5 | | |
| --- | --- | --- | --- | --- | --- | --- | --- | --- | --- | --- | --- | --- | --- | --- | --- | --- | --- | --- | --- |
| Variable | b |  | se |  | b |  | se |  | b |  | se |  | b |  | se |  | b |  | se |
| Male (1 = yes) | 0.17 |  | (0.16) |  | 0.27 |  | (0.15) |  | 0.35 | ^*^ | (0.15) |  | 0.07 |  | (0.16) |  | 0.30 |  | (0.16) |
| Age (ref. 50 to 64 years) |  |  |  |  |  |  |  |  |  |  |  |  |  |  |  |  |  |  |  |
| 65 to 74 years | -0.16 |  | (0.23) |  | 0.29 |  | (0.21) |  | 0.36 |  | (0.20) |  | -0.06 |  | (0.24) |  | -0.08 |  | (0.20) |
| 75 years and older | 0.17 |  | (0.36) |  | 0.26 |  | (0.34) |  | 0.55 |  | (0.30) |  | 0.06 |  | (0.28) |  | 0.16 |  | (0.30) |
| Education (ref. no or primary) |  |  |  |  |  |  |  |  |  |  |  |  |  |  |  |  |  |  |  |
| Secondary | 0.10 |  | (0.37) |  | -0.40 |  | (0.49) |  | -0.30 |  | (0.38) |  | -0.63 |  | (0.46) |  | -0.39 |  | (0.40) |
| Higher | -0.16 |  | (0.36) |  | -0.91 |  | (0.49) |  | -0.63 |  | (0.39) |  | -1.13 | ^*^ | (0.45) |  | -0.60 |  | (0.40) |
| Retired (1 = yes) | 0.04 |  | (0.24) |  | -0.43 | ^*^ | (0.22) |  | -0.54 | ^*^ | (0.21) |  | -0.03 |  | (0.23) |  | -0.43 |  | (0.23) |
| Eq. disposable household income (ref. < €1,500.00) | |  |  |  |  |  |  |  |  |  |  |  |  |  |  |  |  |  |  |
| €1,500.00–€1,999.99 | -0.07 |  | (0.23) |  | -0.69 | ^**^ | (0.25) |  | -0.05 |  | (0.25) |  | -0.35 |  | (0.27) |  | -0.58 |  | (0.31) |
| €2,000.00–€2,999.99 | -0.50 | ^*^ | (0.23) |  | -0.96 | ^***^ | (0.25) |  | -0.27 |  | (0.26) |  | -0.92 | ^**^ | (0.28) |  | -0.96 | ^**^ | (0.32) |
| ≥ €3,000.00 | -0.62 | ^*^ | (0.24) |  | -1.02 | ^***^ | (0.28) |  | -0.22 |  | (0.27) |  | -0.52 |  | (0.30) |  | -0.92 | ^**^ | (0.35) |
| Migration background (1 = yes) | 0.50 |  | (0.39) |  | -0.32 |  | (0.39) |  | -0.25 |  | (0.26) |  | -0.52 |  | (0.30) |  | -0.36 |  | (0.30) |
| Having long-term health problems (1 = yes) | 0.57 | ^*^ | (0.23) |  | -0.03 |  | (0.20) |  | -0.20 |  | (0.19) |  | 0.34 |  | (0.22) |  | 0.26 |  | (0.19) |
| Being disabled (1 = yes) | 0.10 |  | 0.24 |  | 0.01 |  | (0.22) |  | 0.45 | ^*^ | (0.21) |  | -0.08 |  | (0.21) |  | 0.04 |  | (0.20) |
| Constant | 2.06 | ^***^ | (0.60) |  | 4.42 | ^***^ | (0.62) |  | 3.32 | ^***^ | (0.63) |  | 3.94 | ^***^ | (0.695) |  | 4.48 | ^***^ | (0.96) |
| Sigma_u | 1.55 |  |  |  | 1.48 |  |  |  | 1.44 |  |  |  | 1.54 |  |  |  | 1.67 |  |  |
| Sigma_e | 3.73 |  |  |  | 4.01 |  |  |  | 3.94 |  |  |  | 3.92 |  |  |  | 4.00 |  |  |
| Wald chi² | 117.01 |  |  |  | 131.79 |  |  |  | 116.62 |  |  |  | 156.46 |  |  |  | 118.86 |  |  |
| p-value | 0.000 |  |  |  | 0.000 |  |  |  | 0.000 |  |  |  | 0.000 |  |  |  | 0.000 |  |  |
| R² | 3.3% |  |  |  | 3.9% |  |  |  | 3.3% |  |  |  | 3.8% |  |  |  | 3.2% |  |  |
| Respondents | 800 |  |  |  | 781 |  |  |  | 827 |  |  |  | 761 |  |  |  | 763 |  |  |
| Vignettes | 5,600 |  |  |  | 5,467 |  |  |  | 5,789 |  |  |  | 5,327 |  |  |  | 5,341 |  |  |

*Note*: Based on a multilevel regression (GLS) with robust standard errors. Tested with controls for design effects (i.e., vignette position and dummy variables for vignette set).

^*^ p < 0.05, ^**^ p < 0.01, ^***^ p < 0.001

## **Supplementary Figure 1.** Visualization of the relative importance of the dimension levels based on the estimated coefficients (and 95% confidence interval) for males (n= 426) and females (n = 374)

Male

Female

*Note*: Based on a multilevel regression (GLS) with robust standard errors. The coefficients were estimated including controls for design effects (i.e., vignette position and dummy variables for vignette set).

## **Supplementary Figure 2.** Visualization of the relative importance of the dimension levels based on the estimated coefficients (and 95% confidence interval) for respondents aged 50 to 64 years (n= 382), 65 to 74 years (n= 331) and 75 years or older (n = 87)

50 to 64

65 to 74

75 or more

*Note*: Based on a multilevel regression (GLS) with robust standard errors. The coefficients were estimated including controls for design effects (i.e., vignette position and dummy variables for vignette set).
